# World Stroke Day — October 29, 2014

**Published:** 2014-10-24

This year on World Stroke Day (October 29), the World Stroke Organization is launching a global campaign focusing on women and stroke. More women than men die from stroke each year (1). Stroke is the second leading cause of death for persons aged >60 years and the third leading cause of disability-adjusted life-years worldwide (2,3). In the United States, each year approximately 795,000 persons have a stroke (4). High blood pressure is the leading risk factor for stroke (1).

CDC is working to help promote stroke awareness and prevention in multiple ways, including the WISEWOMAN program, the Paul Coverdell National Acute Stroke Program (PCNASP), and the Million Hearts initiative. WISEWOMAN helps women with little or no health insurance reduce their risk for heart disease, stroke, and other chronic diseases. PCNASP funds 11 states to improve the quality of care and transition of care from first contact with emergency medical services through in-hospital care and transition to next care provider. Million Hearts, which is co-led by CDC and the Centers for Medicare & Medicaid Services, aims to prevent 1 million heart attacks and strokes by 2017.

CDC encourages everyone to know the signs and symptoms of stroke and to call 9-1-1 right away if they think they or someone else might be having a stroke. Getting fast treatment is important to prevent death and disability from stroke. Healthy lifestyle changes and medication also can reduce the risk for stroke for some persons. Additional information regarding World Stroke Day is available at http://www.strokeassociation.org/STROKEORG/General/World-Stroke-Day-2012_UCM_444999_SubHomePage.jsp. Additional information regarding CDC’s efforts to address stroke is available at http://www.cdc.gov/stroke/cdc_addresses.htm, and additional information about Million Hearts is available at http://millionhearts.hhs.gov/index.html.
